# The Effectiveness and Safety of Moxibustion for Treating Knee Osteoarthritis: A PRISMA Compliant Systematic Review and Meta-Analysis of Randomized Controlled Trials

**DOI:** 10.1155/2019/2653792

**Published:** 2019-12-17

**Authors:** Ting Yuan, Jun Xiong, Xue Wang, Jun Yang, Yunfeng Jiang, Xiaohong Zhou, Kai Liao, Lingling Xu

**Affiliations:** ^1^Jiangxi University of Traditional Chinese Medicine, Nanchang, China; ^2^The Affiliated Hospital of Jiangxi University of Traditional Chinese Medicine, Nanchang, China

## Abstract

**Background:**

Knee osteoarthritis (KOA) seriously affects people's life. Therefore, it has already become a worldwide health concern. Moxibustion has a significant clinical effect on KOA. This systematic review and meta-analysis is performed to renew previous studies and strictly evaluate the quality of RCT and thus test the effect and safety of moxibustion for KOA.

**Objective:**

To evaluate the effectiveness and safety of moxibustion treatment for alleviating pain and improving lower limb function for patients with KOA.

**Materials and Methods:**

CNKI (1979∼2019), CBM (1979∼2019), VIP (1989∼2019), WF (1998∼2019), PubMed (1966∼2019), Embase (1980∼2019), Cochrane Library, and Web of Science (1900∼2019) were all retrieved by a computer from their inception to June 02, 2019, replenished by manual retrieval of relevant bibliographies. Randomized controlled trials (RCTs) were included if moxibustion was compared to western medicine or negative control (placebo moxibustion or no treatment or UC) for treating KOA. The primary outcomes were the total effect and the Western Ontario and McMaster Universities Osteoarthritis Index (WOMAC scale). The secondary outcomes include VAS, Symptom score, Lysholm score, and Lequesne score. RCTs were collected, and the quality of evidence was evaluated by using the Jadad scale and Cochrane risk assessment tools. We used RevMan5.3.0 software for meta-analysis.

**Results:**

A total of 39 RCTs were included, including 3293 patients. In the assessment of the quality, the evidence differs from low to high based on the Cochrane Bias Evaluation Tools and Jadad scale. Fourteen trials were of high quality, ten were of moderate quality, and 15 were of low quality. Therefore, the quality of the included studies was moderate. In this study, there were 66.67% of the literature, and only 17.95% of the literature correctly reported randomized grouping and allocation of hidden information, respectively. In adverse reactions, only 13 trials included were reported in the study. The main adverse reactions of moxibustion are burns and blisters, whereas the western medicine group was in epigastric discomfort. As for the total effective rate, the meta-analysis of 27 RCTs showed a significant effect of moxibustion VS western medicine (RR = 1.20, 95% CI = 1.16 to 1.25, *I*^2^ = 45%, *P*=0.007); as for the WOMAC scale, the subgroup meta-analysis of 13 trials showed that there was a statistically significant effect of moxibustion VS western medicine (MD = −11.08, 95% CI = −11.72 to −10.44, *I*^2^ = 98%, *P* < 0.00001) and 2 trials on moxibustion VS negative control (MD = −8.38, 95% CI = −12.69 to −4.06, *I*^2^ = 0%, *P*=0.77); as for the VAS score, the meta-analysis of 6 trials showed that there was a significant effect of moxibustion VS western medicine (MD = −2.12, 95% CI = −2.30 to −1.93, *I*^2^ = 98%, *P* < 0.00001); as for the symptom score, the meta-analysis of 7 trials showed that there was a significant effect of moxibustion VS western medicine (MD = −0.81, 95% CI = −1.24 to −0.37, *I*^2^ = 50%, *P*=0.06); as for the Lysholm score, the meta-analysis of 5 trials showed that there was a significant effect of moxibustion VS western medicine (MD = 7.61, 95% CI = 6.04 to 9.17, *I*^2^ = 95%, *P* < 0.00001); and as for the Lequesne score, the meta-analysis of 3 trials showed that there was a significant effect of moxibustion VS western medicine (MD = 3.29, 95% CI = 2.93 to 3.65, *I*^2^ = 99%, *P* < 0.00001).

**Conclusion:**

Moxibustion treatment for KOA is more effective than the positive control (western medicine) or negative control (placebo moxibustion or no treatment or UC), and there were fewer adverse reactions to moxibustion. Due to the universally low quality of the eligible trials, it still needs further large-scale and high-quality randomized controlled trials to verify the effectiveness and safety of moxibustion in the treatment of KOA.

## 1. Introduction

Knee osteoarthritis (KOA) is the most usual and frequent disease of arthritis caused by subchondral bone hyperplasia, which destroys joints and generates deformations increasingly, affecting the functions of knee joints severely, also named as proliferative osteoarthritis or degenerative arthritis, or hypertrophic arthritis [1, 2]. The prominent clinical features of KOA are pain, stiffness, swelling, joint cavity effusion, and motor dysfunction [2]. The etiology of KOA is varied, mainly caused by meniscus injury. The meniscus is composed of fibrous cartilage, one inside and one outside, and is located in the joint space of the knee, which acts as a buffer against shock and cartilage protector [3]. KOA is a primary reason for pain and functional limitation, which reduces the patients' quality of life (QOL) significantly [4]. In China, KOA mainly occurs in middle-aged and old people, with the symptomatic prevalence of 10.3% and 5.7%, respectively [5]. KOA and hip OA constitute the major global health burden together, ranking 11th topmost among global contributors to disability and 38th topmost in disability-adjusted life years [6]. Today, although most guidelines recommend the multimodality drug and nondrug methods as the treatment for KOA [7], long-term use can cause significant adverse reactions, such as gastrointestinal irritation and bleeding, perforated ulcers, hepatic toxicity, and renal toxicity. Hence, nonpharmacological therapy is frequently used in China, including complementary and alternative medicines, for instance, of moxibustion [8].

Moxibustion, a traditional Chinese medicine (TCM) treatment, is made of moxibustion material, mainly moxibustion leaves, produces heat to stimulate specific acupuncture points or parts of the body surface, regulates the function of visceral organs by stimulating meridian qi, and to achieve the purpose of treating diseases [9]. And it is usually used in patients with KOA for its representative function of nondrug intervention in TCM [10]. Although there are many clinical trials and studies on moxibustion in the treatment of KOA, systematic review and meta-analysis of moxibustion or moxibustion combined with western medicine in the treatment of KOA is still insufficient, and a lot of clinical studies have confirmed that western medicine has obvious side effects and is not conducive to long-term use. Moxibustion has fewer side effects and higher safety. Therefore, this study compared the effectiveness and safety of moxibustion in the treatment of KOA in accordance with the method of systematic review and meta-analysis.

Presently, there were six systematic reviews and meta-analysis of moxibustion for KOA [11–16]. Among them, there were four Chinese articles [11–14] and two [15, 16] English articles. The authors of five articles were from China and one from South Korea. Two articles in Chinese literature compared the effectiveness of moxibustion and other therapies for KOA [11, 12]. Although there were two papers studying the effectiveness of moxibustion in the treatment of KOA, there are also some disadvantages, for instance, too few included samples, low quality, or long publication time [13, 14]. Although two of the English articles also studied the effectiveness of moxibustion for KOA [15, 16], only one searched the English database [15], and the other had deficiencies such as too little sample size and few outcome indicators [16]. Hence, the aim of this study is to renew, improve, and strictly evaluate the quality of RCTs to test the effectiveness and safety of moxibustion in the treatment of KOA and better guide the clinical practice of acupuncture and moxibustion.

## 2. Methods

### 2.1. Protocol Register

We conducted this systematic review and meta-analysis strictly according to the PRISMA (The Preferred Reporting Items for Systematic Review and Meta-analysis) statement [17] (S1 PRISMA Checklist). Moreover, we published this protocol in PROSPERO 2015 CRD42015016920 in advance. It is available from http://www.crd.york.ac.uk/PROSPERO/display_record.php?ID=CRD42015016920.

### 2.2. Types of Studies

Only randomized clinical trials (RCTs) were included in this systematic review and meta-analysis. There was no limitation on patients' age, gender, course of the disease, syndrome type, and source of cases. The study subjects had recognized diagnostic criteria and therapeutic effectiveness criteria, and they were all diagnosed with KOA. The commonly used diagnostic criteria in China followed the guidelines for diagnosis and treatment of osteoarthritis, guidelines for clinical research of new traditional Chinese medicine, and criteria for diagnosis and therapeutic effectiveness of TCM diseases and syndromes revised by the orthopedic branch of the Chinese medical association in 2007. The diagnostic criteria of knee arthritis of the American College of Rheumatology (ACR) were followed abroad. There were no language restrictions.

### 2.3. Exclusion Criteria

Duplicate detection and publications, non-moxibustion intervention studies, expert experience, case report, theoretical studies, experimental studies, unclear diagnostic criteria, abstract and conference articles, meaningless interventions in the control group, and incomplete data of the results will be excluded.

### 2.4. Types of Interventions

We included the studies that used various forms of moxibustion (e.g., direct or indirect moxibustion, heat-sensitive moxibustion, gingpao moxibustion, warm needling, or salt-separated moxibustion) as the single therapy or as the main part of a combination treatment with other interventions (e.g., western medicine). The trials, whether the control group received the consistent concomitant treatments as the treatment group, would also be included. And the trials that moxibustion was used as an ancillary treatment would be excluded.

### 2.5. Types of Comparators

We included comparators of no treatment, placebo moxibustion, or related standard treatment for KOA, including western medicine and conventional therapies. If the design of the trial does not consider the evaluation of the effectiveness of moxibustion, the trial will be excluded (e.g., the control group was treated with unproven efficacy therapy, or two different forms of moxibustion were compared) or if they adopted comparators between treatments whose expected effectiveness was consistent to that of moxibustion (e.g., acupuncture).

### 2.6. Types of Outcome Measures

#### 2.6.1. Major Outcomes

The primary outcomes included the total effect and the WOMAC scale. According to the total WOMAC score of the patient, the effectiveness was evaluated concerning the Nimodipine method, namely, index improvement rate = [(pretreatment symptom score-posttreatment symptom score) ÷ pretreatment symptom score] × 100%. Cure: index improvement rate >75%; remarkable effect: index improvement rate ≥50% and ≤75%; effective: index improvement rate ≥30% and <50%; invalid: index improvement rate <30%. The WOMAC scale was reliable reported pain, stiffness, and function measures for osteoarthritis by the global scale value of the Western Ontario and McMaster Universities Osteoarthritis Index (WOMAC) questionnaire. Pain and function scores were converted to a 0–100 scale using the WOMAC items that assess pain (five items) and function (17 items). Higher scores on the WOMAC indicate worse pain and functional status.

#### 2.6.2. Additional Outcome(s)

The secondary outcomes included VAS, Symptom score, Lysholm score, Lequesne score, and adverse reactions: Visual Analogue scale (VAS) (score range, 0∼10): higher scores indicate worse knee pain; Symptom score: according to the guidelines for clinical research on new Chinese medicine in 2002 on the grading of osteoarthritis symptoms, according to the pain, joint swelling, joint activity, morning stiffness, and TCM syndrome diagnosis criteria for grading; Lysholm score: mainly used to evaluate knee flexion and extension activity, consisting of 8 questions with a score of 0∼100. Score above 95 points is excellent, 94∼85 is good, 84∼65 is fair, and less than 65 is poor; Lequesne score: the knee osteoarthritis severity index score scale was used to evaluate the severity of knee osteoarthritis in patients from the aspects of joint motion pain, night pain, morning stiffness, and daily activities. The score on the score scale was directly proportional to the severity of symptoms; and adverse reactions: the severity, frequency, and duration were observed.

### 2.7. Information Sources

We adopted a comprehensive and exhaustive search strategy, including searching electronic databases, manually searching references, contacting pharmaceutical companies, and lead authors. We searched the following electronic databases: CNKI, CBM, VIP, WF, PubMed, Embase, Web of Science, and the Cochrane Library from their inception to June 2, 2019, without language limitation. On July 2, we conducted a repeat search with the same search strategy to fill in the gaps. We also manually searched the relevant journals and bibliographies. There were no limitations on publication years or publication status.

### 2.8. Search Strategy

The comprehensive search strategy for PubMed is listed in Table 1.

### 2.9. Data Extraction

Firstly, according to the PICOST principle, the standard data extraction table was set up in advance. Before the formal data extraction, two preliminary tests were carried out to ensure the quality of data extraction. Then, two evaluators (Ting Yuan and Jun Yang) read the titles and abstracts back-to-back independently to conduct a preliminary screening of the literature and then read the full text in the same way to determine the final included studies. Whenever possible, an intention-to-treat (ITT) analysis of the missing data was performed. Relevant details included author information, year, sample size, a period of treatment, diagnostic criteria, outcome measures, interventions, comparators, and the lost follow-up situation and then cross-checked the results of the included trials. If there is any difference, the inclusion will be decided by discussion or the third evaluator (Xue Wang). When there is incomplete information in the study, it is necessary to contact the first author to obtain relevant data according to the provisions of the standardized protocol.

### 2.10. Quality Assessment

The evaluation criteria of the Jadad scale were strictly followed [18], and the Cochrane risk assessment tool [19] was used to evaluate the included studies. According to Cochrane Handbook 5.2.0, quality reviews and risk of bias are available. The details are as follows: random sequence generation, allocation concealment, blinding of participants and doctors, blinding of outcome evaluator, incomplete outcome data, selective reporting, and other bias. Each item was classified according to a high, low, or unclear risk of bias that is represented as high (H), low (L), and unclear (U), respectively. The quality evaluation results included in the test were cross-examined by two evaluators (Ting Yuan and Jun Yang), and any differences that were difficult to determine could be solved by discussion or the third evaluator (Xue Wang).

### 2.11. Summary Measures and Data Synthesis

Meta-analysis was performed with RevMan5.3.0 software. The heterogeneity test was conducted between studies, with *P* < 0.1 and *I*^2^ > 50% as the test level. When there was no statistical heterogeneity between studies, the fixed effect model was adopted. In the absence of clinical or methodological heterogeneity, a randomized effect model was used. Continuous variables used mean difference MD; relative ratio (RR) was adopted as the categorical variable, and both effect sizes were expressed as 95% CI. *P* ≤ 0.05 was considered statistically significant. If there was significant clinical heterogeneity between studies, only descriptive analysis was performed. If necessary, sensitivity analysis is selected to test the stability of results, and RevMan5.3.0 software is used to analyze the publication bias.

### 2.12. Risk of Bias across Trials

If the RCTs were more than 10, the funnel plot might be used to detect publication bias of the included trials in this meta-analysis.

### 2.13. Additional Analysis

Subgroup analysis and sensitivity analysis were performed to explore the potential heterogeneity and confounders on outcomes. And the subgroup analysis is predefined in the PROSPERO protocol.

### 2.14. Ethical Statement

There were no ethical approval requirements for this study.

## 3. Results

### 3.1. Search Results

**Figure 1:**
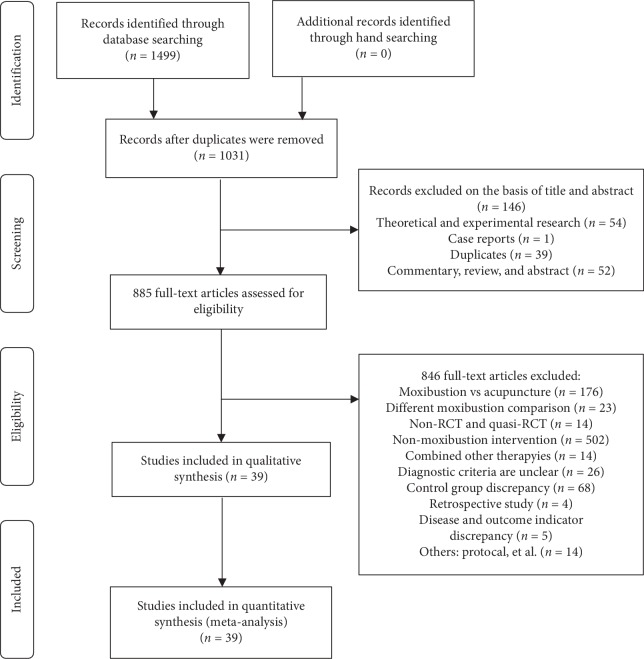
Flow diagram of the study.

A total of 1499 studies were retrieved at the initial search. NoteExpress 2.2.0 software was used for statistical management, and unqualified studies were excluded. At last, a total of 39 eligible trials were included, as shown in Figure 1.

### 3.2. Study Characteristics

The characteristics of all included RCTs are documented. All RCTs were published from 2006 to 2019. There were 1640 patients in the treatment group and 1653 in the control group, respectively. There were four RCTs with three groups, but only two of them met the inclusion and exclusion criteria. Therefore, we only extracted the baseline data of these two groups. The number of patients in each trial varied from 21 to 110. A majority of the patients were in the outpatient or inpatient department. There were 22 RCTs using the ACR (American College of Rheumatology) diagnostic criteria, 8 using the guiding principles of clinical research on new drugs of traditional Chinese medicine and 9 using the guidelines for diagnosis and treatment of osteoarthritis 2007. Besides, the outcome data and other information of each included study are listed in Table 2.

#### 3.2.1. Types of Studies

All of the eligible trials were randomized clinical trials (RCTs) and five were multicenter RCTs [2, 30, 38, 39, 53].

#### 3.2.2. Types of Intervention

Sixteen RCTs [21–24, 26, 29, 32, 37, 40–42, 44, 45, 48, 53] adopt warm-needling moxibustion treatment; ten RCTs [2, 25, 30, 31, 38, 39, 49, 51, 55, 57] adopt moxibustion; two RCTs [28, 50] adopt heat-sensitive moxibustion; two RCTs [27, 54] adopt thunder fire moxibustion; two RCTs [33, 52] adopt herb cake-partitioned moxibustion (HCPM); two RCTs [34, 56] adopt separated aconite cake mild moxibustion (SACM); two RCTs [46, 47] adopt crude herb moxibustion; one RCT [20] adopts three-volt heat-sensitive wheat moxibustion; one RCT [35] adopts gingpao moxibustion; one RCT [36] adopts salt-separated moxibustion; and one RCT [43] adopts Sanqi cake moxibustion.

#### 3.2.3. Types of Control

Thirty-three RCTs [2, 20–25, 27–29, 32–37, 40–48, 50–57] adopt western medicine treatment; four RCTs [30, 31, 39, 49] adopt placebo moxibustion treatment; one RCTs [25] adopt no treatment, and one RCTs [38] adopt UC treatment.

#### 3.2.4. Types of Outcome Measures

Twenty-seven RCTs [2, 20–25, 28, 29, 32–37, 40–42, 44, 45, 48, 50–55, 57] assess the total effective rate. Fifteen RCTs [2, 22–27, 33, 35, 36, 38, 40, 42, 47, 53] used the WOMAC scale to assess the pain and physical function, six RCTs [2, 20, 21, 27, 45, 51] selected the VAS scale, seven RCTs [21, 35, 45–47, 50, 56] selected the Symptom score, five RCTs [21, 22, 32, 45, 51] selected the Lysholm score, and three RCTs [23, 25, 55] selected the Lequesne score to assess pain or symptoms, respectively.

### 3.3. Risk of Bias Assessment

**Figure 2:**
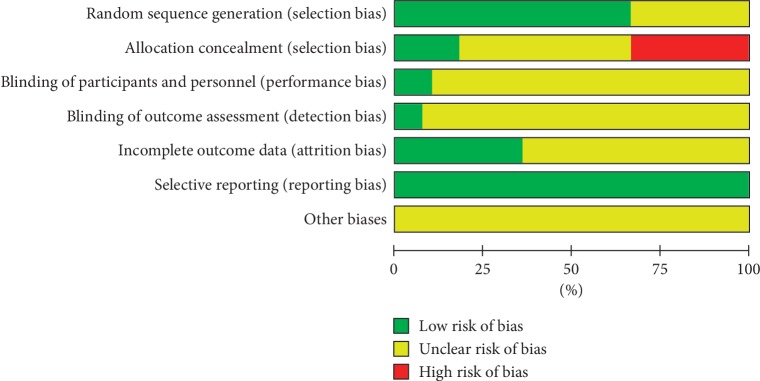
Risk of bias graph.

**Figure 3:**
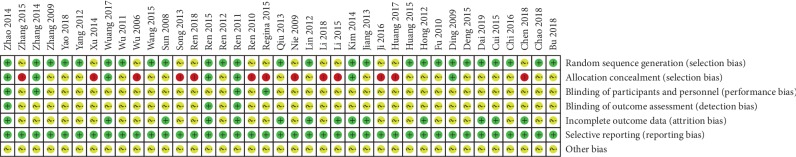
Risk of bias summary.

(1) Randomization: 16 RCTs [2, 20, 21, 28, 32, 33, 36, 42, 44, 45, 48, 50, 51, 53, 55, 56] were randomized by the random number table, 6 RCTs [26, 30, 38–40, 49] were randomized by a computer, 4 RCTs [22, 35, 46, 47] were randomized by draw, and 13 RCTs [23–25, 27, 29, 31, 34, 37, 41, 43, 52, 54, 57] were randomized word only. (2) Allocation hiding: only 7 RCTs [26, 30, 38–40, 49, 53] mentioned proper allocation hiding, 20 RCTs mentioned using random number table or draw random, and 13 RCTs did not mention whether allocation hiding. (3) Blind method: only 2 RCTs [39, 49] implemented the double-blind method, 3 RCTs [30, 31, 40] implemented the single-blind method, the rest of the experiments did not mention whether the blind method was used. (4) Selective report: all studies reported preset outcome indicators; (5) Follow-up and abscission: only 13 RCTs [2, 26, 30, 32, 42, 46, 47, 49, 54, 56] reported the number of cases of abscission and its causes in detail, as shown in Table 3 and Figures 2 and 3.

### 3.4. Quantitative Review and Meta-Analysis

#### 3.4.1. Total Effective Rate

**Figure 4:**
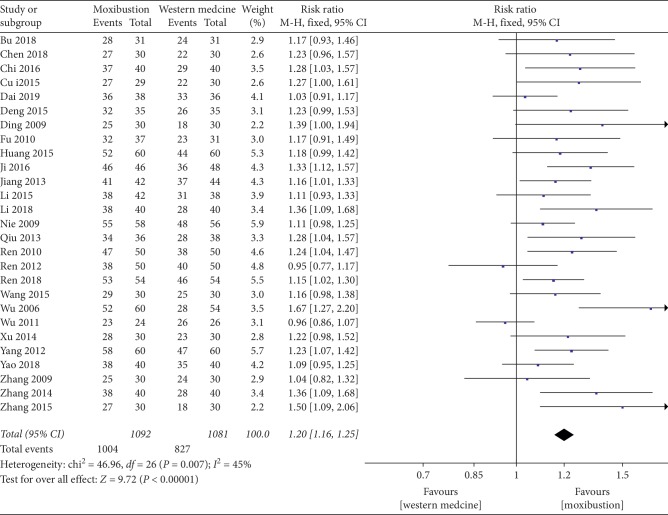
Effects of moxibustion according to the total effective rate.

The forest plot illustrating the results of the meta-analysis for the total effective rate is shown in Figure 4; twenty-seven RCTs compared the effectiveness of moxibustion versus western medicine alone and showed a significant effect of moxibustion on KOA (RR = 1.20, 95% CI = 1.16 to 1.25, *I*^2^ = 45%, *P*=0.007).

#### 3.4.2. WOMAC Scale

**Figure 5:**
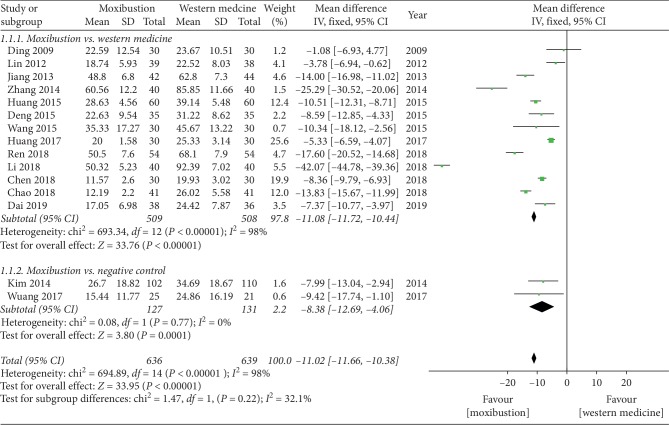
Effects of moxibustion according to the WOMAC scale.

*(1) Moxibustion VS Western Medicine*. The subgroup analysis showed that meta-analysis of the data on using the WOMAC scale (Figure 5), and eight RCTs compared the effectiveness of moxibustion versus western medicine alone and showed a significant effect of moxibustion on KOA (MD = −11.08, 95% CI = −11.72 to −10.44, *I*^2^ = 98%, *P* < 0.00001).

*(2) Moxibustion VS Negative Control*. The subgroup analysis showed that 2 RCTs compared the effectiveness of moxibustion versus negative control and showed a significant effect of moxibustion on KOA (MD = −8.38, 95% CI = −12.69 to −4.06, *I*^2^ = 0%, *P*=0.77).

#### 3.4.3. VAS Score

**Figure 6:**
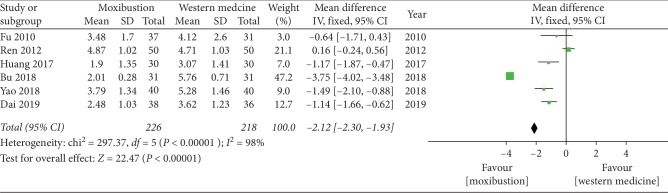
Effects of moxibustion according to the VAS score.

The forest plot illustrating the results of the meta-analysis for the VAS score is shown in Figure 6. Six RCTs compared the effectiveness of moxibustion versus western medicine alone and showed a significant effect of moxibustion on KOA (MD = −2.12, 95% CI = −2.30 to −1.93, *I*^2^ = 98%, *P* < 0.00001).

#### 3.4.4. Symptom Score

**Figure 7:**
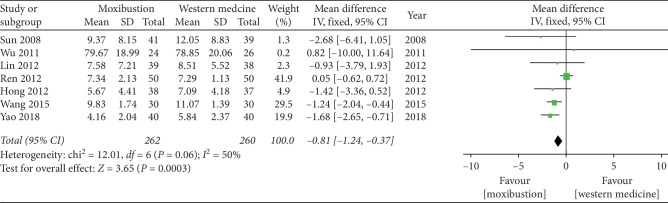
Effects of moxibustion according to the Symptom score.

The forest plot illustrating the results of the meta-analysis for the Symptom score is shown in Figure 7, and seven RCTs compared the effectiveness of moxibustion versus western medicine alone and showed a significant effect of moxibustion on KOA (MD = −0.81, 95% CI = −1.24 to −0.37, *I*^2^ = 50%, *P*=0.06).

#### 3.4.5. Lysholm Score

**Figure 8:**
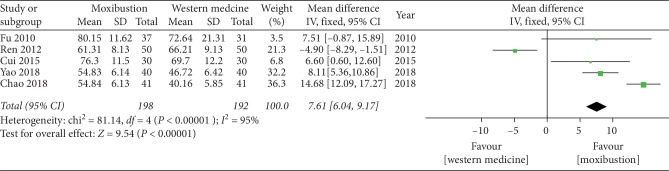
Effects of moxibustion according to the Lysholm score.

The forest plot illustrating the results of the meta-analysis for the Lysholm score is shown in Figure 8. Five RCTs compared the effectiveness of moxibustion versus western medicine alone and showed a significant effect of moxibustion on KOA (MD = 7.61, 95% CI = 6.04 to 9.17, *I*^2^ = 95%, *P* < 0.00001).

#### 3.4.6. Lequesne Score

**Figure 9:**
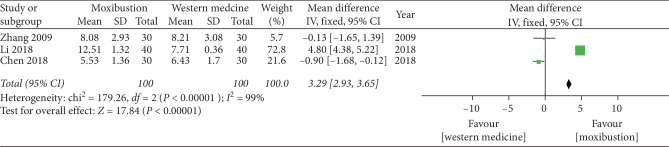
Effects of moxibustion according to the Lequesne score.

The forest plot illustrating the results of the meta-analysis for the Lequesne score is shown in Figure 9. Three RCTs compared the effects of moxibustion versus western medicine alone and showed a significant effect of moxibustion on KOA (MD = 3.29, 95% CI = 2.93 to 3.65, *I*^2^ = 99%, *P* < 0.00001).

#### 3.4.7. Adverse Reactions

**Figure 10:**
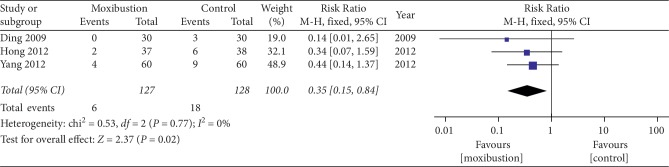
Meta-analysis for adverse events of moxibustion.

Adverse reactions were reported in 13 trials included in the study. Four trials [25, 35, 42, 44] reported that no significant adverse reactions or accidents occurred in each group. Wang et al. [26] reported that 1 case of slight scald occurred in the treatment group. Ren et al. [30] reported that there were 22 patients suffered from blisters of different sizes from the moxibustion. Sit and Zhao [31, 39] reported that ten patients developed skin flushing at the treated sites after real moxibustion. Without any medical measures, the flush disappears naturally within three days. Kim et al. [38] reported that there were 121 AEs in the treatment group. Hong et al. [46] reported that there were 2 cases of blisters in the treatment group and 6 cases of gastrointestinal discomfort in the control group. Qin [47] reported that there were 2 cases of blisters in the treatment group. Yang et al. [48] reported that there were 9 cases of adverse reactions in the control group and 2 cases of minor scald blisters and 2 cases of local pain at the acupuncture site in the treatment group. Ding et al. [53] reported that epigastric discomfort was found in 3 patients in the western medicine group, and there were no adverse events in the treatment group. Therefore, the adverse reactions in the moxibustion group were considered acceptable by the patients, and it did not affect the statistics of the final result data. Only three trials [46, 48, 53] documented the specific number of adverse events of two groups. There was no heterogeneity in the 3 trials (*P*=0.77, *I*^2^ = 0%). The forest plot illustrating the results of the meta-analysis for adverse reactions is shown in Figure 10, and three trials showed no statistical difference between two groups of moxibustion in the treatment of KOA (RR = 0.35, 95% CI = 0.15 to 0.84, *I*^2^ = 0%, *P*=0.77).

### 3.5. Study Heterogeneity

*I* ^2^ values were <50% for the following outcomes: the total effective rate (*I*^2^ = 45%), WOMAC scale when moxibustion VS negative control (*I*^2^ = 0%) and adverse reactions (*I*^2^ = 0%). *I*^2^ values were ≥50% (indicating moderate or substantial heterogeneity) for the following outcomes: WOMAC scale when moxibustion VS western medicine (*I*^2^ = 98%), VAS score (*I*^2^ = 98%), Symptom score (*I*^2^ = 50%), Lysholm score (*I*^2^ = 95%), and Lequesne score (*I*^2^ = 99%).

### 3.6. Publication Bias

**Figure 11:**
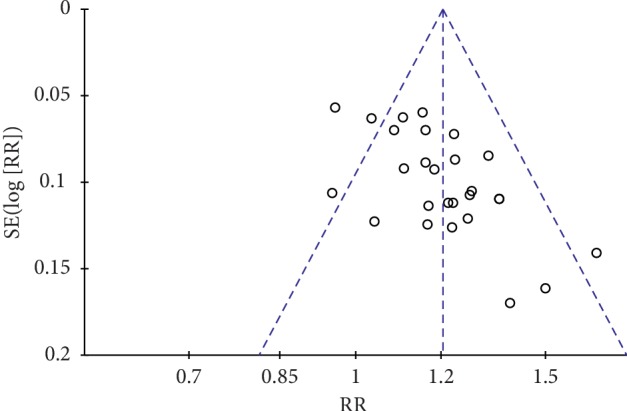
Funnel plot based on total effective rate publication bias.

**Figure 12:**
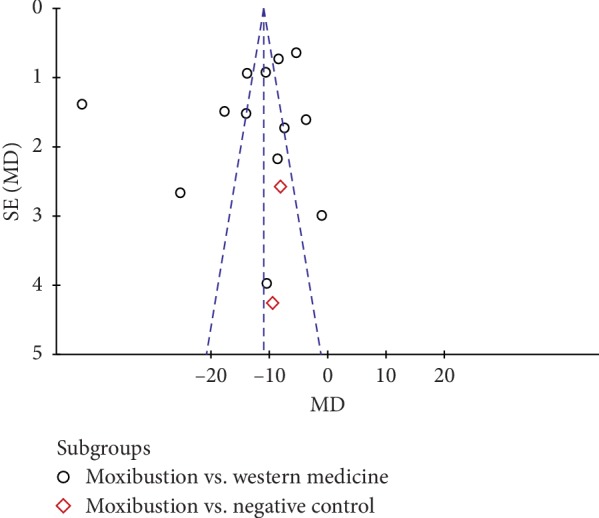
Funnel plot based on WOMAC-scale publication bias.

Based on the total effective rate and WOMAC scale of RevMan5.3.0 software, we used a funnel plot to have a qualitative analysis of publication bias. The distribution of graphical cues was not symmetric, and four points (Figure 11) and eight points (Figure 12) were distributed beyond the funnel, indicating that there might be publication bias in our study that influenced the results of our analysis, as shown in Figures 11 and 12.

### 3.7. Subgroup Analyses

The results of subgroup analysis were summarized for the total effect of moxibustion treatment on KOA. The subgroup analysis showed that there were significant differences within subgroups based on the quality of all included studies and sorts of western medicine and period. *I*^2^ values were <50% and *P* value >0.1 for the following subgroups: quality and period. *I*^2^ values were ≥50% and *P* value <0.1 for the following subgroup: sorts of western medicine. Therefore, the quality of included studies was regarded as the source of methodological heterogeneity and the period was regarded as the source of clinical heterogeneity. On the contrary, because the heterogeneity of western medicine was still high, we changed the fixed effect model into a randomized effect model. The results showed a decrease in heterogeneity (*P*=0.34, *I*^2^ = 12.0%), which indicated that the sorts of western medicine were regarded as the source of statistical heterogeneity, as shown in Table 4.

### 3.8. Sensitivity Analyses

We found that the results of heterogeneity comparing the WOMAC scale on moxibustion VS western medicine, VAS score, and Lysholm score were not significantly reduced by omitting the study sequentially. However, the results of heterogeneity comparing the total effective rate significantly reduced (RR = 1.21, 95% CI = 1.17 to 1.26, *P*=0.16, *I*^2^ = 22%) after excluding the Wu and Xiong [50] study. Therefore, the Wu 2011 [50] study was regarded as the source of heterogeneity. Similarly, the results of heterogeneity comparing symptom score significantly reduced (MD = −1.42, 95% CI = −1.99 to −0.85, *P*=0.95, *I*^2^ = 0%) after excluding the Ren and Li [45] study. Therefore, the Ren and Li [45] study was regarded as the source of heterogeneity. And the results of heterogeneity comparing Lequesne score significantly reduced (MD = −0.74, 95% CI = −1.43 to −0.05, *P*=0.38, *I*^2^ = 0%) after excluding the Li et al. [23] study. Therefore, the Li et al. [23] study was regarded as the source of heterogeneity, as shown in Table 5.

## 4. Discussion

### 4.1. Moxibustion Intervention Mechanism

Moxibustion therapy can reduce cartilage damage and macrophage infiltration, improve local blood circulation in the knee joint by inhibiting the expression of inflammatory factors such as mast cell cyclooxygenase, interleukin-6, and tumor necrosis factor, and repair articular chondrocytes [58–61]. Relevant animal experiments showed that moxibustion could increase the limb pedal strength of knee joints of rats by regulating transformed growth factor and insulin-like growth factor [62]. At present, there are few studies on the definite mechanism of moxibustion in patients with KOA. Therefore, it is necessary to conduct more experimental studies on moxibustion intervention in KOA to better guide clinical practice.

### 4.2. Main Findings of Moxibustion Intervention Effects

The results of this meta-analysis showed a significant total effective rate of moxibustion on KOA (RR = 1.20, 95% CI = 1.16 to 1.25, *I*^2^ = 45%, *P*=0.007). In addition, moxibustion intervention also showed significant differences in WOMAC scores (MD = −11.02, 95% CI = −11.66 to −10.38, *P*=0.22, *I*^2^ = 32.1%), VAS score (MD = −2.12, 95% CI = −2.30 to −1.93, *I*^2^ = 98%, *P* < 0.00001), Symptom score (MD = −0.81, 95% CI = −1.24 to −0.37, *I*^2^ = 50%, *P*=0.06), Lysholm score (MD = 7.61, 95% CI = 6.04 to 9.17, *I*^2^ = 95%, *P* < 0.00001), and Lequesne score (MD = 3.29, 95% CI = 2.93 to 3.65, *I*^2^ = 99%, *P* < 0.00001) in patients with KOA. In general, moxibustion intervention can reduce pain and improve knee symptoms of patients with KOA and has fewer adverse reactions. Therefore, moxibustion treatment for KOA is safe and effective, which is worthy of clinical application.

### 4.3. Quality of Evidence

Among the 39 RCTs were included in this study, containing 3293 patients. The quality of evidence differs from low to high based on the Cochrane Bias Evaluation Tools and Jadad scale. Fourteen trials were of high quality, ten were of moderate quality, and fifteen were of low quality. Therefore, the quality of the included studies was moderate. The inappropriate random method, allocation concealment, and a lack of blinding of most studies exaggerated the results of the outcome measures. In this study, there were 66.67% of the literature and 17.95% of the literature correctly reported randomized grouping and allocation of hidden information, respectively. This could lead to overestimate.

### 4.4. Discussion of Heterogeneity

There was heterogeneity in the effectiveness of moxibustion on the total effective rate of KOA. To explore its source, we adopted subgroup analysis and found that clinical heterogeneity was mainly related to the sorts of western medicine, duration of intervention, and other factors, its methodological heterogeneity was mainly affected by the quality of the included literature and the subjective bias of literature quality evaluation, and the sorts of western medicine was regarded as the source of statistical heterogeneity. Similarly, by changing the research effect model and adopting the sensitivity analysis method, we found that the heterogeneity was significantly reduced after omitting the study of Wu and Xiong [50], Ren and Li [45], and Li et al. [23]. To explore its reasons, it was found that they all have problems such as low quality, the flaw in test design, or small sample sizes. This indicated that the results of this meta-analysis, to a certain extent, were affected by the risk of bias.

### 4.5. Limitations and Advantages

There were several limitations of this systematic review and meta-analysis, as follows:

  Firstly, the evaluation criteria for the total effective rate were inconsistent. There were eight trials using the WOMAC scale, nine trials using guiding principles of clinical research on new drugs of traditional Chinese medicine, two trials using criteria for diagnosis and effectiveness of TCM diseases, and eleven trials using other criteria to evaluate the clinical efficacy of the patients with KOA. Therefore, it might be one of the sources of heterogeneity. However, due to the limitation of the number of included literature, the lack of integrated data and this review did not distinguish effectiveness criteria. Therefore, it is suggested that clinical trials should be strengthened in future studies, and clinical effectiveness should be evaluated by the internationally unified effectiveness evaluation standards. Thus, the results have more authenticity and reliability.
  Secondly, the methodological quality of most included studies was relatively low, and there was a latent risk of bias, to some extent, which weakened the credibility and reliability of the evidence of moxibustion therapy for KOA in this systematic review and meta-analysis. For example, since few control groups were placebo controls, it was difficult to eliminate the placebo effect. Although the random word appeared in all the included literature, only 26 trials described the correct random method. In most of the included trials, allocation concealment and blind method were not clear, which may lead to potential implementation bias and selectivity bias. Most of the included RCTs were conducted in China, and only seven articles were published in English. And the results of the funnel plot indicated potential publication bias.
  Finally, some individual data were incomplete, so we failed to follow the preset subgroup analysis based on variable comparisons. Also, because the necessary data were not available, so patients with either meniscal symptoms or who have degenerative meniscal disease were not discussed as a subgroup within the review. On the contrary, to explore the source of heterogeneity, we performed a subgroup analysis of the total effect based on the quality of all included studies, sorts of western medicine and period.
  While this systematic review and meta-analysis had some limitations, it showed some significant advantages. Most importantly, compared with published systematic review and meta-analysis, literature retrieval in this study was more comprehensive with systematic literature retrieval strategy, and the number of included literature was larger. Therefore, the evidence was more reliable and scientific. In addition, subgroup analysis was performed according to the quality of all included studies, sorts of western medicine and period. And we used more adequate outcome indicators to conduct this meta-analysis. Thus, the stability of our research results was demonstrated. Finally, this systematic review and meta-analysis were performed following the PRISMA statement strictly, and the content was more complete.

## 5. Conclusion

In this systematic review and meta-analysis of RCTs, the effectiveness and safety of moxibustion in the treatment of KOA are positive. But it still needs a well-designed, rigorous, large sample, and multicenter prospective randomized controlled trials on this subject to confirm the validity of the results. In the future, we can also include trials, for example, moxibustion combined with positive controls (e.g., western medicine) VS positive controls (e.g., western medicine) to expand the number of original studies, so as to include higher-quality studies, reduce the influence of publication bias, and improve the credibility of research and better guide clinical practice.

## Figures and Tables

**Table 1:** Search strategy.

Source: PubMed; searched on June 12, 2019	
Search	Query
#1	“knee osteoarthritis” [Ti/Ab] or “knee pain” [Ti/Ab] or “osteoarthritis of knee” [Ti/Ab] or “knee joint osteoarthritis” [Ti/Ab] or “knee arthritis” [Ti/Ab] or “knee gonitis” [Ti/Ab]
#2	“moxibustion” [title/abstract]
#3	“randomized controlled trial” [Ti/Ab] or “clinical trial” [Ti/Ab]
#4	#1 and #2 and #3
#5	“knee osteoarthritis” [MeSH]
#6	“moxibustion” [MeSH]
#7	“randomized controlled trial” [MeSH] or “controlled clinical trial” [MeSH]
#8	#5 and #6 and #7
#9	#4 or #8

**Table 2:** Basic characteristics of eligible RCTs.

Study ID	Sample size T/C	Diagnostic criteria	Intervention		Period (w)	Outcome	Drop out
			Trial group	Control group			
Dai et al. [2]	80 (38/36)	2	Moxibustion	Celecoxib	4/4	Total effective rate, VAS, WOMAC	T : 2, C : 4
Bu et al. [20]	62 (31/31)	2	Three-volt heat-sensitive wheat moxibustion	Futaline emulsion	4/4	Total effective rate, VAS	NR
Yao [21]	80 (40/40)	ACR	Warm-needling moxibustion	Ibuprofen	8/8	Total effective rate, VAS, LKSS, Symptom score	NR
Chao [22]	82 (41/41)	ACR	Warm-needling moxibustion	Ibuprofen	3/3	WOMAC, LKSS	NR
Li et al. [23]	80 (40/40)	ACR	Warm-needling moxibustion	Ibuprofen	2/2	Total effective rate, WOMAC, Lequesne–Mery	NR
Ren [24]	108 (54/54)	ACR	Warm-needling moxibustion	Glucosamine sulfate tablets	20/20	Total effective rate, WOMAC	NR
Chen et al. [25]	60 (30/30)	2	moxibustion	Diclofenac sodium	4/4	Total effective rate, WOMAC, Lequesne–Mery	NR
Wang et al. [26]	50 (25/21)	ACR	Warm-needling moxibustion	No treatment	3/3	Total effective rate, WOMAC	T : 0, C : 4
Huang and Ji [27]	60 (30/30)	2	Thunder fire moxibustion	Celecoxib	5/5	WOMAC, VAS	NR
Chi et al. [28]	120 (40/40/40)	1	Heat-sensitive moxibustion	Sodium hyaluronate	4/4	Total effective rate	NR
Ji [29]	96 (48/48)	ACR	Warm-needling moxibustion	Nabumetone capsules	6/6	Total effective rate	NR
Ren et al. [30]	150 (69/67)	ACR	Moxibustion	Placebo moxibustion	6/6	Non	T : 8, C : 6
Sit et al. [31]	110 (55/55)	ACR	Moxibustion	Placebo moxibustion	6/6	WOMAC	NR
Cui [32]	90 (29/30/30)	2	Warm-needling moxibustion	Glucosamine sulfate tablets	3/3	Total effective rate, WOMAC, Lysholm	T : 1, C : 0
Huang et al. [33]	120 (60/60)	1	HCPM	Diclofenac sodium	4/4	Total effective rate, WOMAC	NR
Zhang and Li [34]	60 (30/30)	1	SACM	Diclofenac sodium	2/2	Total effective rate	NR
Wang [35]	60 (30/30)	2	Gingpao moxibustion	Sodium hyaluronate	8/8	Total effective rate, WOMAC, VAS, Symptom score	NR
Deng et al. [36]	70 (45/45)	2	Salt-separated moxibustion	Ibuprofen	4/4	Total effective rate, WOMAC	NR
Li et al. [37]	80 (42/38)	ACR	Warm-needling moxibustion	Ibuprofen	4/4	Total effective rate	NR
Kim et al. [38]	212 (102/110)	ACR	Moxibustion	UC	5/5	WOMAC	NR
Zhao et al. [39]	110 (55/55)	ACR	Moxibustion	Placebo moxibustion	6/6	WOMAC	NR
Zhang [40]	80 (40/40)	ACR	Warm-needling moxibustion	Diclofenac sodium	3/3	Total effective rate, WOMAC	NR
Xu et al. [41]	60 (30/30)	ACR	Warm-needling moxibustion	Diclofenac sodium	3/3	Total effective rate, HSS	NR
Jiang et al. [42]	90 (42/44)	ACR	Warm-needling moxibustion	Glucosamine sulfate tablets	8/8	Total effective rate, WOMAC, knee flexion	T : 3, C : 1
Song et al. [43]	80 (40/40)	2	Sanqi cake moxibustion	Diclofenac sodium	3/3	Total effective rate, WOMAC	NR
Qiu [44]	74 (36/38)	ACR	Warm-needling moxibustion	Ibuprofen	4/4	Total effective rate	NR
Ren and Le [45]	150 (50/50/50)	1	Warm-needling moxibustion	Sodium hyaluronate	3/3	Total effective rate, VAS, Lysholm, Symptom score	NR
Hong et al. [46]	78 (38/37)	ACR	Crude herb moxibustion	Glucosamine sulfate	12/6	Total effective rate, Symptom score	T : 2, C : 1
Lin [47]	64 (31/31)	ACR	Crude herb moxibustion	Glucosamine sulfate	12/6	Total effective rate, WOMAC, Symptom score	T : 1, C : 1
Yang et al. [48]	120 (60/60)	ACR	Warm-needling moxibustion	Celecoxib	4/4	Total effective rate, VAS, Lequesne	NR
Ren et al. [49]	65 (31/28)	ACR	Moxibustion	Placebo moxibustion	6/6	WOMAC	T : 2, C : 4
Wu and Xiong [50]	50 (24/26)	1	Heat-sensitive moxibustion	Sodium hyaluronate	3/3	Total effective rate, Symptom score	NR
Fu et al. [51]	68 (37/31)	ACR	Moxibustion	Sodium hyaluronate	24/24	Total effective rate, VAS, Lysholm	NR
Ren et al. [52]	100 (50/50)	1	HCPM	Diclofenac sodium	3/3	Total effective rate	NR
Ding et al. [53]	90 (30/30/30)	ACR	Warm-needling moxibustion	Ibuprofen	2/2	Total effective rate, WOMAC	NR
Nie et al. [54]	116 (58/58)	1	Thunder fire moxibustion	Ibuprofen	5/5	Total effective rate	NR
Zhang [55]	60 (30/30)	2	Moxibustion	Diclofenac sodium	2/2	Total effective rate, Lequesne	NR
Sun et al. [56]	60 (29/27)	1	SACM	Diclofenac sodium	3/3	Total effective rate, Symptom score	T : 1, C : 3
Wu et al. [57]	114 (60/54)	ACR	Moxibustion	Diclofenac sodium	3/3	Total effective rate	NR

Note: 1 = guiding principles of clinical research on new drugs of traditional Chinese medicine; 2 = guidelines for diagnosis and treatment of osteoarthritis 2007/2010; ACR = American College of Rheumatology; UC = regimen performed according to own intention; NR = not reported; AKS = American Knee Society Knee score; VAS = Visual Analogue scale; LKSS = Lysholm knee score; HSS = hospital for special surgery knee score; SF-36 scale = short form 36 questionnaire; WOMAC = Western Ontario and McMaster Universities Osteoarthritis Index; HCPM = herb cake-partitioned moxibustion; SACM = separated aconite cake mild moxibustion.

**Table 3:** Risk of bias in the included RCTs.

Study	Random sequence generation	Allocation concealment	Blinding		Outcome data integrity	Selective outcome reporting	Other biases
			Patient/doctor blinding	Outcome assessor blinding			
Dai et al. [2]	Random number table	Uncertain	Uncertain	Uncertain	Low risk	Uncertain	Uncertain
Bu et al. [20]	Random number table	Uncertain	Uncertain	Uncertain	Uncertain	Uncertain	Uncertain
Yao [21]	Random number table	Uncertain	Uncertain	Uncertain	Uncertain	Uncertain	Uncertain
Chao [22]	Draw random	Uncertain	Uncertain	Uncertain	Uncertain	Uncertain	Uncertain
Li et al. [23]	Random word	High risk	Uncertain	Uncertain	Uncertain	Uncertain	Uncertain
Ren [24]	Random word	High risk	Uncertain	Uncertain	Uncertain	Uncertain	Uncertain
Chen et al. [25]	Random word	High risk	Uncertain	Uncertain	Low risk	Uncertain	Uncertain
Wang et al. [26]	Computer random	Low risk	Uncertain	Uncertain	Low risk	Uncertain	Uncertain
Huang and Ji [27]	Random word	High risk	Uncertain	Uncertain	Uncertain	Uncertain	Uncertain
Chi et al. [28]	Random number table	Uncertain	Uncertain	Uncertain	Uncertain	Uncertain	Uncertain
Ji [29]	Random word	High risk	Uncertain	Uncertain	Uncertain	Uncertain	Uncertain
Ren et al. [30]	Computer random	Low risk	Uncertain	Low risk	Low risk	Uncertain	Uncertain
Sit et al. [31]	Random word	High risk	Low risk	Uncertain	Uncertain	Uncertain	Uncertain
Cui [32]	Random number table	Uncertain	Uncertain	Uncertain	Low risk	Uncertain	Uncertain
Huang et al. [33]	Random number table	Uncertain	Uncertain	Uncertain	Uncertain	Uncertain	Uncertain
Zhang and Li [34]	Random word	High risk	Uncertain	Uncertain	Uncertain	Uncertain	Uncertain
Wang [35]	Draw random	Uncertain	Uncertain	Uncertain	Uncertain	Uncertain	Uncertain
Deng et al. [36]	Random number table	Uncertain	Uncertain	Uncertain	Uncertain	Uncertain	Uncertain
Li et al. [37]	Random word	High risk	Uncertain	Uncertain	Low risk	Uncertain	Uncertain
Kim et al. [38]	Computer random	Low risk	Uncertain	Uncertain	Low risk	Uncertain	Uncertain
Zhao et al. [39]	Computer random	Low risk	Low risk	Low risk	Low risk	Uncertain	Uncertain
Zhang [40]	Computer random	Low risk	Single blind	Uncertain	Uncertain	Uncertain	Uncertain
Xu [41]	Random word	High risk	Uncertain	Uncertain	Uncertain	Uncertain	Uncertain
Jiang et al. [42]	Random number table	Uncertain	Uncertain	Uncertain	Low risk	Uncertain	Uncertain
Song et al. [43]	Random word	High risk	Uncertain	Uncertain	Uncertain	Uncertain	Uncertain
Qiu [44]	Random number table	Uncertain	Uncertain	Uncertain	Low risk	Uncertain	Uncertain
Ren and Li [45]	Random number table	Uncertain	Uncertain	Uncertain	Uncertain	Uncertain	Uncertain
Hong et al. [46]	Draw random	Uncertain	Uncertain	Uncertain	Low risk	Uncertain	Uncertain
Lin [47]	Draw random	Uncertain	Uncertain	Uncertain	Low risk	Uncertain	Uncertain
Yang et al. [48]	Random number table	Uncertain	Uncertain	Uncertain	Uncertain	Uncertain	Uncertain
Ren et al. [49]	Computer random	Low risk	Low risk	Low risk	Low risk	Uncertain	Uncertain
Wu and Xiong [50]	Random number table	Uncertain	Uncertain	Uncertain	Uncertain	Uncertain	Uncertain
Fu et al. [51]	Random number table	Uncertain	Uncertain	Uncertain	Uncertain	Uncertain	Uncertain
Ren et al. [52]	Random word	High risk	Uncertain	Uncertain	Uncertain	Uncertain	Uncertain
Ding et al. [53]	Random number table	Low risk	Uncertain	Uncertain	Uncertain	Uncertain	Uncertain
Nie et al. [54]	Random word	High risk	Uncertain	Uncertain	Uncertain	Uncertain	Uncertain
Zhang [55]	Random number table	Uncertain	Uncertain	Uncertain	Uncertain	Uncertain	Uncertain
Sun et al. [56]	Random number table	Uncertain	Uncertain	Uncertain	Low risk	Uncertain	Uncertain
Wu et al. [57]	Random word	High risk	Uncertain	Uncertain	Uncertain	Uncertain	Uncertain

**Table 4:** Subgroup analysis of the total effective rate for each variable.

Variable	No. of trials	No. of participants		RR (95% CI)	*P* value	*I* ^2^ value
		Events	Total			
Quality						
High quality	6	367	433	1.23 [1.13, 1.33]	0.60	0%
Low quality	21	1464	1740	1.20 [1.15, 1.25]		

Western medicine						
Celecoxib	2	174	194	1.15 [1.04, 1.27]	0.08	52.4%
Glucosamine sulfate	3	210	241	1.22 [1.10, 1.34]		
Sodium hyaluronate	5	302	358	1.09 [1.00, 1.19]		
Ibuprofen	7	474	558	1.20 [1.11, 1.29]		
Diclofenac sodium	8	521	654	1.29 [1.19, 1.40]		

Period						
Two weeks	4	203	260	1.31 [1.14, 1.49]	0.31	16.9%
Three weeks	7	458	563	1.23 [1.13, 1.33]		
Four weeks	9	626	740	1.19 [1.12, 1.27]		
Eight weeks	3	205	226	1.13 [1.04, 1.23]		

*P* value and *I*^2^ value test for heterogeneity between subgroups.

**Table 5:** Summary of sensitivity analysis for the total effective rate, Symptom score, and Lequesne score.

	RR fluctuation	*P* fluctuation	*I* ^2^ fluctuation
Total effective rate	(1.20 to 1.21)	(0.007 to 0.16)	(45% to 22%)

	MD fluctuation	*P* fluctuation	*I* ^2^ fluctuation
Symptom score	(−0.81 to −1.42)	(0.06 to 0.95)	(50% to 0%)
Lequesne score	(−0.74 to 3.29)	(<0.00001 to 0.38)	(99% to 0%)

## Abbreviations

KOA: Knee osteoarthritis
CI: Confidence interval
MD: Mean difference
RR: Risk ratio
RCT: Randomized controlled trial
UC: Regimen performed according to own intention.

## Additional Points

S1 PRISMA Checklist. The PRISMA Checklist of this meta-analysis. PRISMA 2009 checklist.doc.
